# Impact of Phytochemicals on Viability and Cereulide Toxin Synthesis in *Bacillus cereus* Revealed by a Novel High-Throughput Method, Coupling an AlamarBlue-Based Assay with UPLC-MS/MS

**DOI:** 10.3390/toxins13090672

**Published:** 2021-09-21

**Authors:** Markus Kranzler, Elrike Frenzel, Veronika Walser, Thomas F. Hofmann, Timo D. Stark, Monika Ehling-Schulz

**Affiliations:** 1 Institute of Microbiology, Department of Pathobiology, Vetmeduni Vienna, Veterinärplatz 1, 1210 Vienna, Austria; markus.kranzler@vetmeduni.ac.at (M.K.); Elrike.Frenzel@brillhygiene.com (E.F.); 2 Food Chemistry and Molecular Sensory Science, Department of Molecular Life Sciences, Technical University of Munich, Lise-Meitner-Str. 34, 85354 Freising, Germany; veronika.walser@tum.de (V.W.); thomas.hofmann@tum.de (T.F.H.); timo.stark@tum.de (T.D.S.)

**Keywords:** *Bacillus cereus*, cereulide, food additives, high-throughput micro-scale method

## Abstract

Due to its food-poisoning potential, *Bacillus cereus* has attracted the attention of the food industry. The cereulide-toxin-producing subgroup is of particular concern, as cereulide toxin is implicated in broadscale food-borne outbreaks and occasionally causes fatalities. The health risks associated with long-term cereulide exposure at low doses remain largely unexplored. Natural substances, such as plant-based secondary metabolites, are widely known for their effective antibacterial potential, which makes them promising as ingredients in food and also as a surrogate for antibiotics. In this work, we tested a range of structurally related phytochemicals, including benzene derivatives, monoterpenes, hydroxycinnamic acid derivatives and vitamins, for their inhibitory effects on the growth of *B. cereus* and the production of cereulide toxin. For this purpose, we developed a high-throughput, small-scale method which allowed us to analyze *B. cereus* survival and cereulide production simultaneously in one workflow by coupling an AlamarBlue-based viability assay with ultraperformance liquid chromatography–mass spectrometry (UPLC-MS/MS). This combinatory method allowed us to identify not only phytochemicals with high antibacterial potential, but also ones specifically eradicating cereulide biosynthesis already at very low concentrations, such as gingerol and curcumin.

## 1. Introduction

*B. cereus* is responsible for an increasing number of food-borne diseases resulting from food-borne infections and intoxications [1,2,3]. Food-borne infections often lead to gastrointestinal symptoms, evoked by various enterotoxins produced by re-germinated bacteria in the small intestine, while intoxications are caused by the emetic toxin cereulide preformed in foods [4,5]. In foods associated with diarrheal syndrome, commonly 10^5^–10^8^ *B. cereus* cells or spores per gram are found [6,7,8]. However, the actual toxic potential of *Bacillus cereus* is highly strain dependent [9], which might explain why occasionally lower amounts have been reported from food-borne *B. cereus* infections.

The 1.2 kDa peptide toxin cereulide [D-*O*-Leu D-Ala L-*O*-Val D-Val]_3_ is synthesized by a non-ribosomal peptide synthetase (NRPS) encoded by the *ces* operon located on the mega-plasmid pCER270, which shares its backbone with the anthrax toxin encoding pX01 plasmid of *Bacillus anthracis* [10,11,12]. Severe cases of cereulide intoxication, including multi-organ failure and fatalities, have been reported [13,14,15,16]. Due to its small size of 1.2 kDa and chemical properties, cereulide (by contrast to bacterial cells or spores) cannot be removed by filtration or inactivated by heating or cooking during food production and processing. It is also not possible to destroy the peptide by enzymatic digestion, hydrolysis or extreme pH changes [17,18]. Hence, once preformed in food contaminated with emetic *B. cereus,* cereulide cannot be eliminated prior to consumption. The storage of high-risk foods at room temperature may especially lead to the enrichment of cereulide [15]. Thus, the prevention of toxin formation in food production and processing, as well as during food storage, is of utmost importance. 

Reports on cereulide synthesis-inhibiting compounds that could be legally used in foods according to GRAS policies are rather scarce due to the limitations of current methods for rapid and easy screening of cereulide in food matrices. Thus, we developed a high-throughput, small-scale method, which combines the detection of *B. cereus* viability and cereulide production by coupling an AlamarBlue-based viability assay with ultraperformance liquid chromatography–mass spectrometry (UPLC-MS/MS), using a microtiter plate format to assess the inhibitory potentials of commonly used food additives, extracts derived from industrial product admixtures, as well as select secondary compounds from plants with reported antibacterial activity. With this study, we aim to open new avenues for preventing cereulide formation in foods by means of natural, non-hazardous, and low-dosed phytochemicals.

## 2. Results and Discussion

### 2.1. Development of a Micro-Scale Assay to Simultaneously Test the Effect of Food Ingredients on Growth and Cereulide Toxin Production in Emetic B. cereus

**Figure 1:**
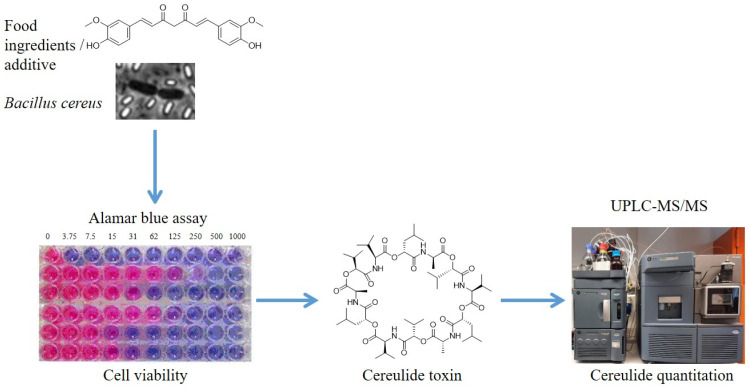
**Impact of phytochemicals on cereulide synthesis & bacterial growth revealed by a novel combinatory method.** Reduction of the viability and cereulide production of the emetic *Bacillus cereus* by application of phytochemicals. Viability was determined by measuring fluorescence in an AlamarBlue assay, and cereulide was quantified, after pooling of samples and ethanolic extraction, via UPLC-MS/MS.

Extrinsic factors, such as temperature, pH, salt, and food matrices have been reported to significantly affect cereulide toxin synthesis in emetic *B. cereus* (for review see [15]), hence it is not possible to deduce the risk of cereulide production from growth rates [19]. A broadscale survey, using an in-situ bioluminescence reporter strain, shed some light onto the modulating effect of food on cereulide production and allowed the analyzed food matrices to be assigned to three risk classes related to their risk of cereulide production [14]. However, since suitable screening methods are lacking to test substances for their impact on the growth of emetic *B. cereus* and their modulating effect on cereulide production simultaneously, information on the influence of specific food ingredients or additives on cereulide production is still very limited. In this study, we aimed to establish a micro-scale method that allows the effects of food ingredients and additives on vegetative growth of emetic *B. cereus* and cereulide formation to be assessed simultaneously in one workflow (see Figure 1).

To evaluate the suitability of this assay for our purpose, we carried out a pilot trial using a selection of food ingredients and additives provided by the dairy industry. In line with results from the work of Collins and Franzblau [20], the microplate AlamarBlue assay allowed us to successfully measure the influence of commonly used food ingredients and additives on the growth of emetic *B. cereus*. No effects or only minimal inhibitory effects were observed for: acetylated distarch adipate (modified corn starch), gelatin, and sodium alginate. These substances also showed no effects or only weak effects at the highest used concentration (2.5%), while carrageenan, pectin and yeast powder with 38% NaCl showed some growth inhibitory effects at the highest concentration used. A strong effect on cell viability was found for: extracts from herb mixture/walnuts/pepper/dried onions, *S*-allyl cysteine, manniflavanone, epicatechin, caffeic acid, DKP cyclo (Pro-Val/Ala-Gly), rutin trihydrate and vanillin. The latter compounds led to growth inhibition at concentrations between 0.0031–1.25% (Figure S1). Next, we investigated whether the bacterial cultures from the AlamarBlue assay could be subsequently used for cereulide toxin analysis by UPLC-MS/MS.

**Figure 2:**
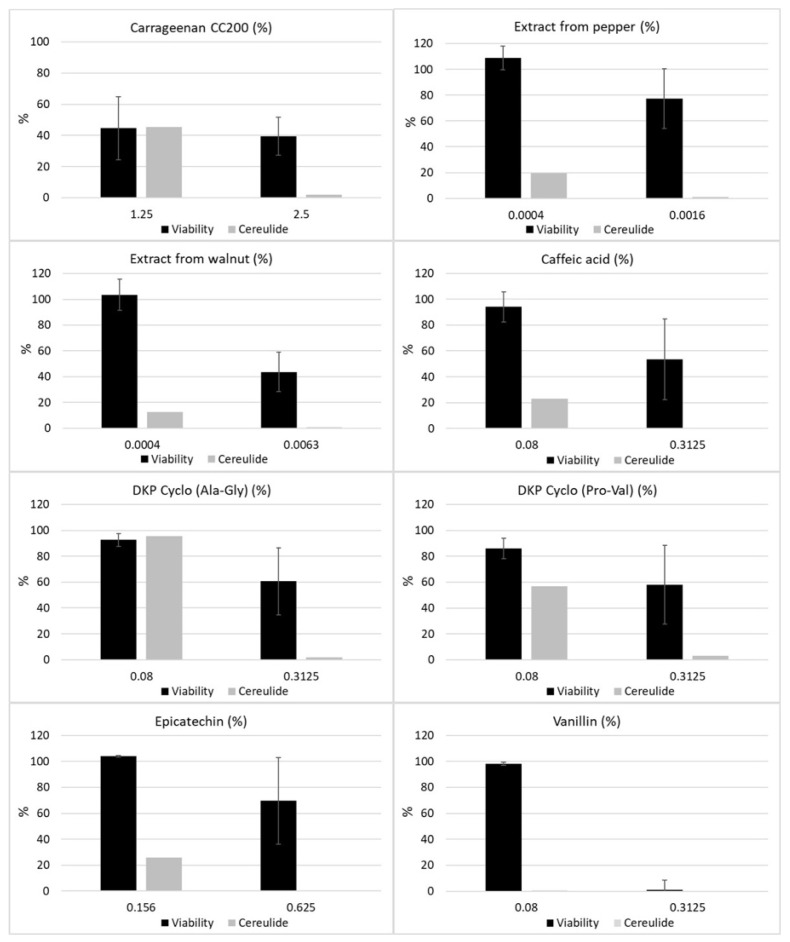
**Inhibition of the growth and cereulide biosynthesis of emetic *B. cereus* by selected food ingredients, extracts and pure substances, as revealed by the novel combinatory microscale assay** (see Figure 1). An AlamarBlue assay was employed to determine the viability of the emetic reference strain *B. cereus* F4810/72 and UPLC-MS/MS was used for quantitation of cereulide after pooling of samples and ethanolic extraction as described in the Materials and Methods Section.

Therefore, the cultures from the microtiter plates were transferred to 1.5 mL tubes and cereulide was extracted following a slightly modified protocol established previously [21]. The relative amounts of cereulide from a subset of tested ingredients that inhibited growth of emetic *B. cereus* in the AlamarBlue assay were determined by UPLC-MS/MS as described previously [22]. Notably, some of the tested ingredients showed a stronger inhibitory effect on cereulide production than on bacterial growth (see Figure 2), of which the most effective were the pure substances caffeic acid and vanillin, belonging to the classes of hydroxycinnamic acids and benzene derivatives, respectively.

These results underline that the risk for cereulide toxin production cannot be deduced from sole growth measurements. A specific inhibitory effect on cereulide synthesis independent of growth inhibition has been reported previously for long chain polyphosphates [23]. Thus, it is tempting to speculate that other food ingredients might differentially modulate cereulide synthesis as well. Identification of such substances could help to develop targeted strategies to prevent cereulide toxin production in the food production and processing chain. Therefore, we focused next on assaying pure substances used in food production and processing.

### 2.2. Selection of Phytochemicals to Be Tested in the Novel Combinatory Micro-Scale Assay

Based on the results of the initial screening of the inhibitory effects of commonly used food ingredients on the growth and toxin production of emetic *B. cereus* (see Figure 2 and Figure S1), a panel of chemically-related molecules belonging to secondary plant compounds, such as terpenes and polyphenols, including derivatives from benzene, cinnamic acid and vitamins, was defined to search for molecules that repress cereulide synthesis and/or bacterial growth (see Table 1). Benzene derivatives were included since they can be found naturally in various plants, fruits, nuts, herbs, and fungi. Some of these compounds are routinely used in food processing and preservation, such as benzoic acid (preservative agent E 210), sodium benzoate (preservative agent E 211), vanillin, anisaldehyde, anisole and *p*-anisic acid (flavoring agents) [24,25,26,27,28]. Capsaicin, curcumin, and gingerol are widespread spices in Indian and Asian cuisine [29,30,31,32]. Terpenes (or isoprenoids) are found in nearly all plant species and have been widely used in the food industry, pharma industry, and also as pesticides due to their broad chemical diversity [33]. Further, terpenes are the main components of essential oils, which have been used in various fields and are also reported as antibacterial agents [34,35]. For instance, monoterpenes with known antimicrobial effects are thymol and its isomer, carvacrol, as well as menthol and eucalyptol [35,36,37,38]. Hydroxycinnamic acid derivatives, such as benzene derivatives, are phenolic acids and are found widespread in diverse fruits, vegetables, and crops. Known agents used in the food industry are caffeic acid, its ester chlorogenic acid, sinapinic acid, ferulic acid, and coumarin [39,40]. Retinol (vitamin A1), *α*-tocopherol (vitamin E), phylloquinone (vitamin K1), menaquinone (vitamin K2), and menadione (vitamin K3) have been classified as vitamins despite their heterogenous chemical structures. Retinol, a terpenoid, is an essential vitamin for animals and humans and is found in meat and plants [41]. *α*-tocopherol, which is produced only by plants, acts as an antioxidant [42]. The K vitamins have important roles in the coagulation cascade and, additionally, in photosynthesis (phylloquinone) and the electron transport chain (menaquinone, menadione) [43]. The occurrence of these compounds in plant-based foods used by the food industry as flavoring agents, preserving agents, or anthraquinone dyes, constitutes the rationale for their selection. An overview of the substances included in this study is provided in Table 1.

### 2.3. Screening of the Phytochemical Panel by Means of the Novel Micro-Scale Alamarblue & UPLC-MS/MS Assay for Inhibitory Substances

The majority of the substances (32 out of 40) tested in our newly established assay, described above, showed an inhibiting effect on both the vegetative growth of *B. cereus* and cereulide production (Table 2). The minimal inhibitory concentration (MIC) ranged from ≤0.001% to 4%. Substances belonging to the group of benzene derivatives and vitamins showed the highest growth inhibitor potential. Five out of the thirteen benzene derivates included in the test panel (capsaicin, curcumin, [8]-gingerol, juglone and xanthohumol) and two out of the five vitamins included in the test panel (menadione and retinol) were categorized as strong inhibitors (MIC ≤ 0.01%), while MICs for monoterpenes ranged from 0.02% (carvacrol and thymol) to 4% (eucalyptol and *α*-phellandrene), and MICs for hydroxycinnamic acids ranged from 0.1% (*p*-coumaric acid and ferulic acid) to 1% (coumarin). In general, the MICs were in accordance with data from literature, underlining the suitability of the small-scale AlamarBlue assay for our screening [34]. For instance, similar to our study, eucalyptol has been reported to possess rather weak antimicrobial activity [44] and phylloquinone (vitamin K1) as well as menaquinone (vitamin K2) have been described to have no antibacterial effects [45]. By contrast, due to its membrane permeability, menadione (vitamin K3) has been described as a highly potent antimicrobial against *Staphylococcus aureus*, *Pseudomomas aeruginosa*, and *Escherichia coli*, with MICs between 64–128 µg/mL [45,46], which matches our current results for *B. cereus.* However, although it is a highly potent antimicrobial, menadione is not permitted as a food additive, due to its side effects and toxicity, which may arise at 1000-fold levels of the required daily dose of vitamin K (60–80 µg/day per adult person) [47].

Notably, the addition of certain substances resulted in a significant decrease of cereulide production (<15%), but did not have a negative impact on bacterial viability (>85%) (see Table 2). When applied in lower concentrations, a specific cereulide inhibitory effect was also observed for some compounds, which inhibited growth at high concentrations. Some substances, especially those belonging to the class of monoterpenes (e.g., citral, myrcene, and nerol), showed even a growth promoting tendency but still a strong cereulide inhibitory effect, highlighting that some phytochemicals specifically impact toxin biosynthesis. These results, which are in line with our previous study showing that long chain polyphosphates have a specific inhibitory effect on cereulide synthesis [23], foster the hypothesis that certain food ingredients and phytochemicals have unexplored potential as targeted strategies to prevent cereulide toxin production in the food production and processing chain. Furthermore, an inhibitory effect of polyphosphates on aflatoxins production by *Aspergillus* spp. and botulinum toxicity of *Clostridium botulinum* was reported previously [48,49].

### 2.4. Identification of Phytochemicals with Inhibitory Potential against B. cereus Growth and Cereulide Biosynthesis

The strongest inhibitory effect was observed for xanthohumol, with an MIC of 6 µg/mL. Similarly, Cermak et al. reported a comparable strong antibacterial effect of this hop-deriving compound against *Bacteroides fragilis* and *Clostridium perfringens* (MIC of 10–60 µg/mL) [50]. Among the benzene derivatives, [8]-gingerol was identified as an additional strong growth inhibitor, showing an MIC of 10 µg/mL, whereas a sublethal concentration of 5 µg/mL sharply decreased cereulide synthesis without affecting the growth behavior significantly. This indicates a dual action mode of [8]-gingerol, influencing both cell viability and cereulide biosynthesis specifically. Data from literature indicate that the antimicrobial activity of gingerol depends on the alkyl modification and length of the side chain, although with contradictory results. Hiserodt and coworkers reported of a lower MIC of [10]-gingerol, compared to [6]- and [8]-gingerol, for *Mycobacterium*, whereas Park et al. postulated higher efficacy of [12]-gingerol than [10]-gingerol for gram-negative periodontal bacteria [31,51]. In particular, [10]-gingerol exhibited an MIC between 6–14 µg/mL and [12]-gingerol an MIC between 15–30 µg/mL [31], which is consistent with the MICs for [8]-gingerol determined for *B. cereus* in our current work. A similar dual mode of action was observed for vanillin, with complete growth inhibition at 2.5 mg/mL and specific repression of cereulide synthesis at 0.8 mg/mL. It has been reported previously that the antibacterial activity of vanillin is linked to its interactions with the bacterial plasma membrane, leading to disturbances in ion gradients, pH homeostasis and inhibition of respiratory enzymes [27]. Moreover, vanillin was shown to bind on the minor groove of the DNA molecule [52], which might explain its specific inhibition of cereulide synthesis observed in our current study. Curcumin was also amongst the most effective compounds inhibiting the growth of emetic *B. cereus* with an MIC of 70 µg/mL. Its inhibitory effect has been described against Gram-negative and -positive bacteria through the formation of membrane pores, which is similar to the mode of action of certain antibiotics and also of capsaicin [53,54]. Furthermore, its non-toxicity and use as a food ingredient renders curcumin [55] as a suitable inhibitory substance to prevent cereulide intoxication. At lower concentrations, curcumin showed, like [8]-gingerol and vanillin, a specific inhibitory effect on cereulide production (see Table 2). 

The most efficient monoterpenes were carvacrol and thymol with an MIC of 0.2 mg/mL (0.02%). This fits with the results of Gallucci and coworkers, who showed that carvacrol exhibited a higher efficiency than thymol, to a minor extent, presumably due to the different position of the hydroxyl group at the phenol ring [56]. Also, Xu et al. (2008) reported a concentration of 0.2 mg/mL of carvacrol or thymol, for inhibition of the growth of *E. coli* [38]. For *B. cereus*, recent studies have reported MICs of 0.007 mg/mL [34] and 0.625 mg/mL [37] for thymol. The cis-trans isomers geraniol and nerol, and its product citral, showed similar results in inhibiting growth and cereulide production. Geraniol and citral revealed an MIC of 0.4 mg/mL, and nerol an MIC of 1 mg/mL, while a higher MIC for citral was reported for *Cronobacter sakazakii* (0.27–0.54 mg/mL) [57]. The latter study showed that the antimicrobial activity of citral is based on cell membrane damage and hyperpolarization, which is similar to curcumin. Notably, myrcene was found to completely repress cereulide biosynthesis at concentrations of 0.5–1% while rather promoting growth of emetic *B. cereus* at these concentrations (Table 2). It was previously reported that myrcene has no inhibitory effect against the growth of *E. coli*, *S. aureus* and *B. cereus* [56]. Thus, the complete repression of cereulide by myrcene, independent of any growth inhibition, highlights again the potential of phytochemicals to be used as a targeted strategy to prevent cereulide toxin formation in food production and processing.

Of the four classes of substances included in this study, the hydroxycinnamic acid derivatives generally showed the highest MICs (1–10 mg/mL). However, similar to the other classes, substances specifically inhibiting cereulide biosynthesis were found among hydroxycinnamic acid derivatives. In particular, caffeic acid inhibited cereulide production at 0.25 mg/mL and 0.5 mg/mL without having significant effects on growth (Table 2). Due to its binding affinity to the minor DNA groove, thereby interfering with transcriptional regulators [58], it is tempting to speculate that it exerts its negative action on cereulide biosynthesis on a transcriptional level. In addition, caffeic acid has been shown to enhance the effect of certain antibiotics against *S. aureus*. The reported MICs (0.2–1 mg/mL) are comparable to those of our current study on *B. cereus* [59], which also makes it a promising candidate for preventing cereulide production in foods.

## 3. Conclusions

Our newly developed combinatory micro-scale assay allowed us to simultaneously identify compounds with inhibitory potential against the growth of emetic *B. cereus* and against cereulide toxin biosynthesis in one workflow. We identified natural, hazard-free compounds, which are able to completely prevent bacterial multiplication and cereulide formation at a concentration of 0.01% and even below. Overall, the most potent substances that could potentially be used in food production and processing were found in the class of benzene derivatives. Moreover, our combinatory approach revealed different modes of action. The majority of substances, such as xanthohumol or thymol, inhibit growth and consequently cereulide synthesis at a certain concentration. However, several substances, such as vanillin or caffeic acid, affect cereulide biosynthesis more stringently than cell viability. Thus, future research should focus on elucidating the different modes of action of phytochemicals described in this work to pave the way for novel strategies to prevent food-borne intoxications by emetic *B. cereus*.

## 4. Materials and Methods

### 4.1. Bacterial Strains 

The emetic reference strain *B. cereus* F4810/72 (also designated AH187) [60] was used in the microplate-based AlamarBlue assays and for subsequent cereulide quantification via UPLC-MS/MS. 

### 4.2. Food Ingredients and Phytochemicals

An overview of the food ingredients and additives (*n* = 30) obtained from the food industry and used for the initial screening of substances modulating cereulide synthesis is given in Table S1. Based on the results from the initial screening, a panel of phytochemicals (*n* = 40) including benzene derivatives, monoterpenes, hydroxycinnamic acids and vitamins, was generated and purchased from Sigma Aldrich, (St. Louis, MO, USA) (see Table 1). The substances were dissolved in 99.9% ethanol, except sodium benzoate (dissolved in dH_2_O) and menaquinone (dissolved in dimethyl sulfoxide; DMSO), according to the manufacturer’s instruction, to prepare stock solutions. The following substances were only available in liquid formulation (>95%): 4-anisaldehyde, anisole, benzaldehyde, (*S*)-(+)-carvone, (*R*)-(-)-carvone, citral, cuminaldehyde, eucalyptol, geraniol, menthol, nerol, *α*-phellandrene, sabinene, *α*-tocopherole. Working solutions were obtained by serial dilutions of stock solutions or by evaporation using a “SpeedVac miVac Duo Plus” vacuum centrifuge (Genevac Ltd., Ipswich, UK). 

### 4.3. Cell Viability Testing with Microscale AlamarBlue Assay

Via preliminary tests, the approximate efficacy of each substance was assayed using twofold serial dilutions. Three concentrations of each substance (not inhibiting, partially inhibiting, and completely inhibiting) were tested in the newly developed AlamarBlue viability assay as described below. All substances were tested at least two times in independent experiments, including each substance in duplicate in each experiment.

The emetic reference strain *B. cereus* F4810/72 was pre-cultured on PC agar for 24 h at 30 °C. Cells from three to five different colonies were homogenized in LB medium and adjusted to a McFarland density of 0.5, equivalent to 5 × 10^6^ CFU/mL. This suspension was diluted to a final cell count of 10^3^ CFU/mL, and 100 µL of the well-dispersed cell inocula were exposed to 100 µL of LB medium containing the respective concentrations of tested substances. Per 96-well plate (Corning Costar Assay plate, black with clear bottom, Sterile, Polystyrene #3904; Sigma-Aldrich), the following controls were included: a sterile control (200 µL LB medium), a growth control (100 µL LB medium plus 100 µL bacterial suspension), and three solvent controls (100 µL LB medium plus 100 µL bacterial suspension and 4, 2, and 1% of ethanol). The outer periphery wells of the plate were filled with 200 µL of sterile double distilled water to prevent evaporation of the medium from the inner wells. The 96-well plates were incubated for 16 h at 30 °C under vigorous shaking (600 rpm) in a microplate incubator with a heated lid (Grant-bio ThermoShaker PHMP-4, Thermo Fisher Scientific, Waltham, MA, USA). To assess the cell viability, the tetrazolium-based redox dye solution AlamarBlue (Thermo Fisher Scientific, Waltham, MA, USA) was diluted to 1:10 with dH_2_O, and 50 µL was added to each well of the plate. After two minutes of incubation, the fluorescence resulting from the reduction of AlamarBlue by metabolically active and/or proliferating cells was measured with a microplate reader (SpectraMax M3, Molecular Devices, San Jose, CA, USA) at 585 nm emission after excitation at 555 nm. To calculate cell viability, background subtraction was performed on all test and the positive control wells (PC) by subtracting the mean background fluorescence units (FU) measured from the negative control (NC) wells. The cell viability was then calculated according to the following formula: (mean test well FU/mean PC well FU) × 100 [20]. After read-out, the plates were stored at −20 °C until cereulide was extracted as described below.

### 4.4. Cereulide Extraction and Quantification via UPLC-MS/MS Analysis

Cereulide was extracted directly from the 96-well plates as follows: After thawing the plates for 20 min at room temperature, the volume of each well containing the same dilution step of test substance was transferred into a 1.5 mL tube (Eppendorf, Hamburg, Germany) and centrifuged for 4 min at 13.000 rpm. The supernatant was discarded and the cereulide was extracted from the pellet with 1 mL HPLC-grade ethanol (99.9%, Honeywell, Seelze, Germany) by shaking at RT overnight. The extracts were centrifuged, filtered (0.22 µm; Phenomenex, Aschaffenburg, Germany), diluted (1:10) with ethanol containing ^13^C_6_-cereulide (100 ng/mL) as internal standard [21] and, after vortexing, directly subjected to UPLC-MS/MS analysis. For calibration, mixtures of cereulide (0.1, 0.5, 1.0, 5.0, 10, 50, 100, 200, 500, and 1000 ng/mL, respectively, in EtOH) and ^13^C_6_-cereulide (100 ng/mL) were prepared from standard solutions and analyzed in triplicates via UPLC-MS/MS. Using the peak area (A) ratios of analyte to internal standard against the concentration (c) ratios of analyte to internal standard for each solution, and applying linear regression, the calibration curve was obtained (origin excluded). The equation for the calibration curve was gained from the quantifier mass transitions of cereulide (*m/z* 1170.7 → 357.2) and ^13^C_6_-cereulide (*m/z* 1176.7 → *m/z* 358.2), resulting in the following: y = 3.4714 x − 0.0117 with y = c (cereulide)/c (^13^C_6_-cereulide), and x = A (cereulide)/A (^13^C_6_-cereulide), and R^2^ = 0.9998.

### 4.5. Ultraperformance Liquid Chromatography—Mass Spectrometry (UPLC-MS/MS)

The mass spectrometric analysis was performed as described previously using a Waters Xevo TQ-S mass spectrometer (Waters, Manchester, UK) aligned with an Acquity UPLC i-class core system (Waters, Milford, MA, USA) containing a binary solvent manager, column oven, and sample manager [22].

2 µL-aliquots of the samples were applied in the UPLC-MS/MS system which was fitted with a 2.1 × 150 mm, 1.7 µm, UPLC CSH C18 column (Waters, Manchester, UK). Device set up and operation were conducted according to literature, with a flow rate of 0.7 mL/min and a temperature of 55 °C [61]. Chromatography was performed with HCOONH_4_ (10 mmol, 0.1% HCOOH, solvent A), and MeCN (0.25% HCOOH, solvent B). The gradient was started at 85% B, within 8.0 min increased to 95% B, within 0.1 min increased to 99% B, held isocratically for 0.9 min, within 0.1 min decreased to 85% B, and followed by 0.9 min re-equilibration at 85% B [61].

Measurements were performed in positive electrospray ionization (ESI) mode and quantitative calibration mode applying ion source parameters according to literature [61]: capillary voltage +3.6 kV, sampling cone 50 V, source offset 35 V, source temperature 150 °C, desolvation temperature 650 °C, cone gas 250 L/h, desolvation gas 1100 L/h, collision gas flow 0.15 mL/min and nebulizer gas flow 7.0 bar. Calibration of the mass spectrometer was performed with phosphoric acid (0.1% in MeCN) from *m/z* 40–1963. The UPLC-MS/MS device (Xevo TQ-S) was controlled with MassLynx^TM^ 4.1 SCN 813 Software (Waters), and analysis and data processing were accomplished with its subdivision TargetLynx (Waters). The ammonium adducts of the target analytes were detected in the multiple reaction monitoring (MRM) mode. Observation of the mass transitions (given in brackets) for 25 ms delivered the following parameters: cereulide (*m/z* 1170.7 → qualifier: *m/z* 172.2, 314.2; quantifier: *m/z* 357.2), and ^13^C_6_-cereulide (*m/z* 1176.7 → m/z qualifier: 173.2, 316.2; quantifier: *m/z* 358.2). All technical parameters for the MRM mode were applied according to literature [61]. ESI^+^ mass as well as product ion spectra were gained via direct flow infusion with IntelliStart. All MS/MS parameters of the analytes were applied according to literature [61]. 

## Figures and Tables

**Table 1:** Phytochemicals used in this study, with their respective stock solution concentration in 99.9% ethanol.

Benzene Derivatives	Stock Solution	Monoterpenes	Stock Solution	Hydroxycinnamic Acids	Stock Solution
Anisaldehyde	≥97.5% *	Camphor	100% (1 g/mL)	Caffeic acid	5% (50 mg/mL)
		Carvacrol	99% *	Chlorogenic acid	25% (0.25 g/mL)
*p*-Anisic acid	1.7% (0.01 g/mL)	(*R*)-Carvone	98% *	Cinnamic acid	50% (0.5 g/mL)
Anisole	≥99% *	(*S*)-Carvone	96% *	*p*-Coumaric acid	10% (0.1 g/mL)
Benzaldehyde	≥99.5% *	Citral	95% *	Coumarin	50% (0.5 g/mL)
Benzoic acid	50% (0.5 g/mL)	Cuminaldehyde	98% *	Ferulic acid	10% (0.1 g/mL)
Capsaicin	1% (0.01 g/mL)	Eucalyptol	99% *	Rosmarinic acid	12.5% (0.125 g/mL)
Curcumin	0.3% (0.003 g/mL)	Geraniol	98% *	Sinapinic acid	5% (0.05 g/mL)
[8]-Gingerol	0.1% (0.001 g/mL)	Menthol	99% *	**Vitamins**	**Stock solution**
Juglone	1% (0.01 g/mL)	Myrcene	100% *	Menadione	5% (0.05 g/mL)
Salicylic acid	10% (0.1 g/mL)	Nerol	100% *	Retinol	25% (0.25 g/mL)
Sodium benzoate **	10% (0.1 g/mL)	*α*-Phellandrene	≥85% *	Menaquinone	25% (0.25 g/mL)
Vanillin	50% (0.5 g/mL)	Sabinene	75% *	Phylloquinone	≥97% *
Xanthohumol	0.1% (0.01 g/mL)	Thymol	50% (0.5 g/mL)	*α*-Tocopherol	≥96% *

* Substances were obtained as liquid stock solution. ** Solution prepared in dH_2_O.

**Table 2:** **Effect of phytochemicals on viability and cereulide production, as determined by the AlamarBlue assay.** Percentage values (%) of viability and cereulide levels are calculated with reference to the untreated control, in which cereulide was quantified as 1.45 µg/mL (=100%). *: Substances with MICs ≤ 0.1 mg/mL were considered as highly effective. -: non detectable.

Substances leading to growth inhibition (Viability < 5%)			
Substance	MIC (mg/mL)	Cereulide (%)	Viability (%)
**Benzene derivatives**			
Anisaldehyde	5.0	-	-
*p-*Anisic acid	1.7		
Benzaldehyde	5.0		
Benzoic acid	2.5		
Capsaicin *	0.1		
Curcumin *	0.07		
[8]-Gingerol *	0.01		
Juglone *	0.1		
Salicylic acid	1.0	-	-
Sodium benzoate	10.0		1.3 ± 1.1
Vanillin	2.5		-
Xanthohumol *	6 × 10^−3^	0.8	
**Monoterpenes**			
Camphor	10.0	-	0.4 ± 0.1
Carvacrol	0.2		-
(*R*)-Carvone	20.0		-
(*S*)-Carvone	20.0		0.6 ± 0.4
Citral	0.4		1.9 ± 1.0
Cuminaldehyde	2.5		-
Eucalyptol	40.0		0.2 ± 0.0
Geraniol	0.4		-
Menthol	1.0		
Nerol	1.0		
*α*-phellandrene	40.0		0.5 ± 0.2
Thymol	0.2		-
**Hydroxycinnamic acid derivatives**			
Chlorogenic acid	5.0	-	2.1 ± 1.8
Cinnamic acid	2.5		-
*p*-Coumaric acid	1.0		
Coumarin	10.0		
Ferulic acid	1.0		
Rosmarinic acid	2.5		
**Vitamins**			
Menadione	0.02 *	-	-
Retinol	0.05 *	-	
**Substances no/moderate growth inhibitory effect (viability > 85%) but leading to decreased cereulide production (<15%)**			
**Substance**	**MIC (mg/mL)**	**Cereulide (%)**	**Viability** **(%)**
**Benzene derivatives**			
Anisole	40.0	-	131.6 ± 22.9
Curcumin *	0.03	0.1	92.3 ± 28.6
[8]-Gingerol *	5 × 10^−3^	12.6	88.6 ± 4.3
Juglone *	0.05	-	109.0 ± 4.7
Sodium benzoate	5.0	-	117.2 ± 6.3
Vanillin	0.8	-	98.2 ± 1.3
**Monoterpenes**			
Citral	0.2	-	133.5 ± 27.2
	0.1	0.2	155.7 ± 31.4
Myrcene	10.0	-	149.5 ± 3.0
	5.0	-	119.8 ± 7.9
Nerol	0.5	-	173.0 ± 24.3
	0.25	-	127.5 ± 22.2
**Hydroxycinnamic acid derivatives**			
Caffeic acid	0.25	7.8	89.5 ± 9.4
Cinnamic acid	0.5	0.2	94.7 ± 12.0
Ferulic acid	0.5	1.6	89.6 ± 1.3
Rosmarinic acid	0.625	-	83.4 ± 9.1
Sinapinic acid	1.0	9.6	113.1 ± 8.6
**Vitamins**			
Menadione *	2 × 10^−3^	-	87.5 ± 13.3
	2 × 10^−4^	8.1	101.4 ± 33.5
Phylloquinone	10.0	12.8	89.9 ± 12.7
	5.0	12.3	110.0 ± 4.4
Retinol	5 × 10^−4^	11.2	108.8 ± 16.0

## Data Availability

Data is contained within the article or the Supplementary Material.

## Supplementary Materials

The following are available online at https://www.mdpi.com/article/10.3390/toxins13090672/s1, Figure S1: AlamarBlue assay to test the impact of food ingredients on viability of the emetic reference strain *B. cereus* F4810/72, Table S1: Stock solution concentrations of food additives and food ingredients commonly found in dairy-based products that were tested in this study.
