# Supplementary material for: Impact of Phytochemicals on Viability and Cereulide Toxin Synthesis in Bacillus cereus Revealed by a Novel High-Throughput Method, Coupling an AlamarBlue-Based Assay with UPLC-MS/MS
Source: Toxins (Basel). 2021 Sep 21;13(9):672. doi: 10.3390/toxins13090672 (PMC8470179; doi:10.3390/toxins13090672)
Supplement: Supplementary file 1 [file toxins-13-00672-s001.zip › toxins-1377486-supplementary.pdf]

Impact of Phytochemicals on Viability and Cereulide Toxin Synthesis in *Bacillus cereus* Revealed by a Novel High-Throughput Method, Coupling an AlamarBlue-Based Assay with UPLC-MS/MS

Markus Kranzler, Elrike Frenzel, Veronika Walser, Thomas F. Hofmann, Timo D. Stark and Monika Ehling-Schulz

Table S1. Stock solution concentrations of food additives and food ingredients commonly found in dairy-based products that were tested in this study.

Substance[§] / E number	Stock solution
**Food ingredients and extracts**	
Carrageenan 1 (CC200), E407	5% (0.05 g/mL) *
Carrageenan 2, E407	5% (0.05 g/mL) *
Carrageenan with dextrose blend (65-80% & 20-35%)	1% (0.01 g/mL) *
Acetylated distarch adipate 1 (corn starch - hot soluble modified), E1422	10% (0.1 g/mL) *
Acetylated distarch adipate 2 (corn starch - hot soluble modified), E1422	10% (0.1 g/mL) *
Acetylated distarch adipate 3 (corn starch - hot soluble modified), E1422	10% (0.1 g/mL) *
Acetylated distarch adipate 4, (corn starch - hot soluble modified), E1422	10% (0.1 g/mL) *
Extract from dried onions	10% (0.1 g/mL) †
Extract from herb mixture	10% (0.1 g/mL) †
Extract from pepper	10% (0.1 g/mL) †
Extract from walnuts	10% (0.1 g/mL) †
Gelatin 1	10% (0.1 g/mL) *
Gelatin 2	10% (0.1 g/mL) *
Gelatin from pork 1	6% (0.06 g/mL) *
Gelatin from pork 2	1% (0.01 g/mL) *
Pectin (E440)	10% (0.1 g/mL) *
Pectin with sucrose blend (E440i)	6% (0.06 g/mL) *
Sodium alginate (E401)	1.5% (0.015 g/mL) *
Yeast powder with 38% NaCl (TM 1.8%)	10% (0.1 g/mL) *
Yeast powder with 38% NaCl (TM 2.2%)	10% (0.1 g/mL) *
**Pure substances**	
Caffeic acid (3-(3,4-Dihydroxyphenyl)-2-propenoic acid)	1% (0.01 g/mL) †
Caffeine (1,3,7-Trimethyl-1*H*-purine-2,6(3*H*,7*H*)-dione 3,7-Dihydro-1,3,7-trimethyl-1*H*-purine-2,6-dione)	1% (0.01 g/mL) *
Diketopiperazine (DKP) cyclo (Ala-Gly)	1% (0.01 g/mL) †
Diketopiperazine (DKP) cyclo (Pro-Val)	1% (0.01 g/mL) †
(-)-Epicatechin ((2*R*,3*S*)-2-(3,4-dihydroxyphenyl)-3,4-dihydro-2*H*-chromene-3,5,7-triol)	1% (0.01 g/mL) †
Manni-flavanone (biflavanone)	1% (0.01 g/mL) †
Rutin trihydrate (2-(3,4-dihydroxyphenyl)-5,7-dihydroxy-3-[*α*-L-rhamnopyranosyl-(1→6)-*β*-D-glucopyranosyloxy]-4*H*-chromen-4-one)	1% (0.01 g/mL) †
S-Allyl-cysteine ((*R*)-2-Amino-3-prop-2-enylsulfanylpropanoic acid)	1% (0.01 g/mL) *
S-Methyl-cysteine ((2*R*)-2-amino-3-(methylsulfanyl)propanoic acid)	1% (0.01 g/mL) *
Vanillin (4-Hydroxy-3-methoxybenzaldehyde)	10% (0.1 g/mL) *

[§] Ingredients, extracts and pure substances were provided by different food producers. * dissolved in dH_2O; [†] dissolved in 50% EtOH

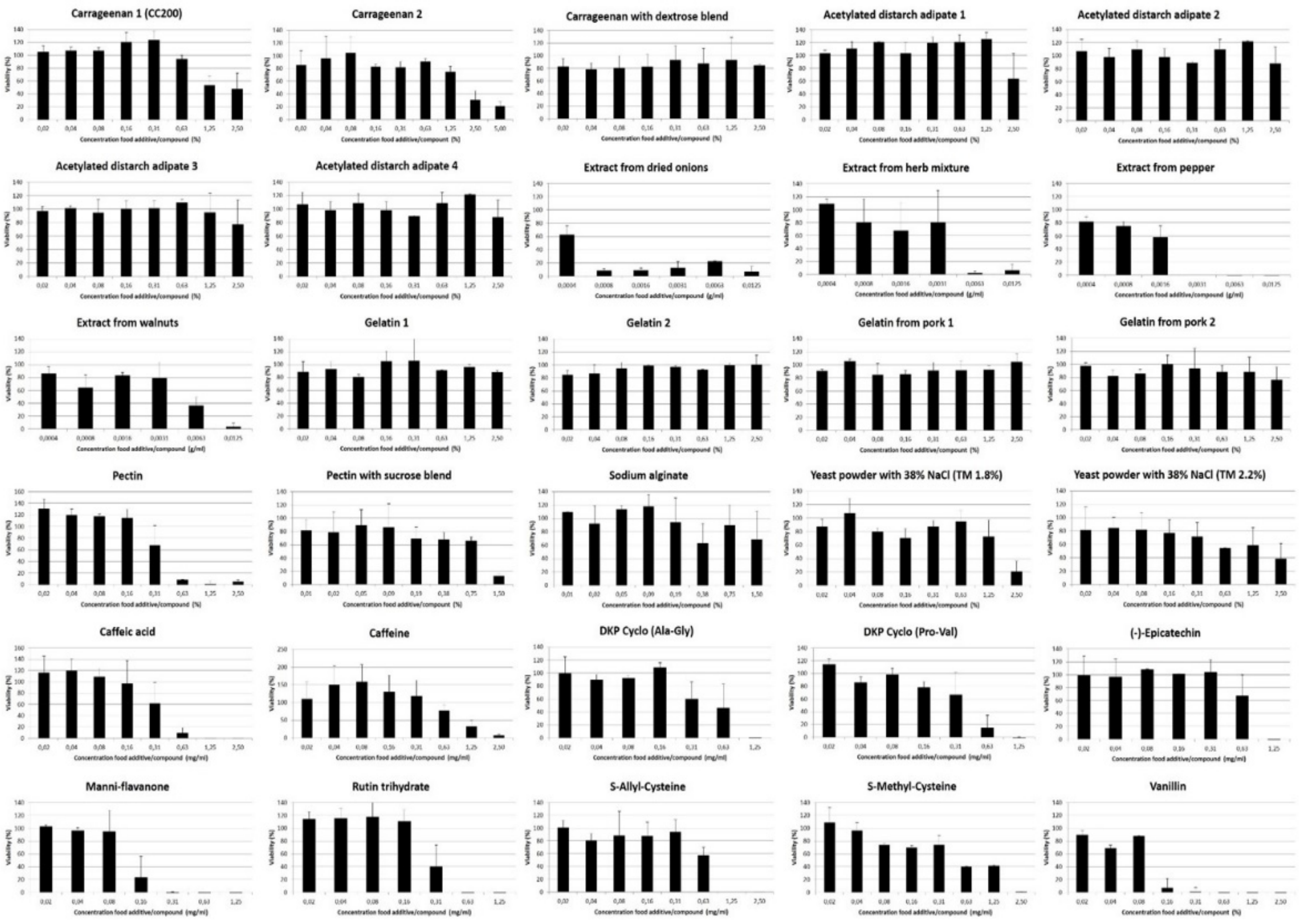

Figure S1. AlamarBlue assay to test the impact of food ingredients on viability of the emetic reference strain *B. cereus* F4810/72. Viability was determined by measuring fluorescence in an AlamarBlue assay as described in the material and method section. Each compound was tested in two biological experiments with each two technical replicates.
